# Machine Listening for OSA Diagnosis: A Bayesian Meta-Analysis

**DOI:** 10.1016/j.chest.2025.04.006

**Published:** 2025-04-11

**Authors:** Benjamin Kye Jyn Tan, Esther Yanxin Gao, Nicole Kye Wen Tan, Brian Sheng Yep Yeo, Claire Jing-Wen Tan, Adele Chin Wei Ng, Zhou Hao Leong, Chu Qin Phua, Maythad Uataya, Liang Chye Goh, Thun How Ong, Leong Chai Leow, Guang-Bin Huang, Song Tar Toh

**Affiliations:** a Department of Otorhinolaryngology−Head & Neck Surgery, Singapore General Hospital, Singapore; b School of Computing and Information, University of Pittsburgh, Pittsburgh, PA; c Yong Loo Lin School of Medicine, National University of Singapore, Singapore; d SingHealth Duke-NUS Sleep Centre, SingHealth, Singapore; e Surgery Academic Clinical Program, SingHealth, Singapore; f Department of Otorhinolaryngology−Head & Neck Surgery, Sengkang General Hospital, Singapore; g Department of Otorhinolaryngology, Faculty of Medicine Siriraj Hospital, Mahidol University, Bangkok, Thailand; h Department of Otorhinolaryngology, Faculty of Medicine, Universiti Malaya, Kuala Lumpur, Malaysia; i Department of Respiratory and Critical Care Medicine, Singapore General Hospital, Singapore; j School of Automation, Southeast University, Nanjing, China; k Key Laboratory of Measurement and Control of Complex Systems of Engineering, Ministry of Education, Nanjing, China; l Mind PointEye, Singapore

**Keywords:** artificial intelligence, computer audition, deep learning, machine learning, sound analysis

## Abstract

**Background:**

Among 1 billion patients worldwide with OSA, 90% remain undiagnosed. The main barrier to diagnosis is the overnight polysomnogram, which requires specialized equipment, skilled technicians, and inpatient beds available only in tertiary sleep centers. Recent advances in artificial intelligence (AI) have enabled OSA detection using breathing sound recordings.

**Research Question:**

What is the diagnostic accuracy of and how can we optimize machine listening for OSA?

**Study Design and Methods:**

PubMed, Embase, Scopus, Web of Science, and IEEE Xplore databases were systematically searched. Two masked reviewers selected studies comparing the patient-level diagnostic performance of AI approaches using overnight audio recordings vs conventional diagnosis (apnea-hypopnea index) using a train-test split or k-fold cross-validation. Bayesian bivariate meta-analysis and meta-regression were performed. Publication bias was assessed by using a selection model. Risk of bias and evidence quality were assessed by using the Quality Assessment of Diagnostic Accuracy Studies-2 and the Grading of Recommendations, Assessment, Development, and Evaluation tools.

**Results:**

From 6,254 records, 16 studies (41 models) trained on 4,864 participants and tested on 2,370 participants were included. No study had a high risk of bias. Machine listening achieved a pooled sensitivity (95% credible interval) of 90.3% (86.9%-93.1%), a specificity of 86.7% (83.1%-89.7%), a diagnostic OR of 60.8 (39.4-99.9), and positive and negative likelihood ratios of 6.78 (5.34-8.85) and 0.113 (0.079-0.152), respectively. At apnea-hypopnea index cutoffs of ≥ 5, ≥ 15, and ≥ 30 events per hour, sensitivities were 94.3% (90.3%-96.8%), 86.3% (80.1%-90.9%), and 86.3% (79.2%-91.1%); and specificities were 78.5% (68.0%-86.9%), 87.3% (81.8%-91.3%), and 89.5% (84.8%-93.3%). Meta-regression identified increased sensitivity for the following: higher audio sampling frequencies, non-contact microphones, higher OSA prevalence, and train-test split model evaluation. Accuracy was equal regardless of home smartphone vs in-laboratory professional microphone recordings, deep learning vs traditional machine learning, and variations in age and sex. Publication bias was not evident, and the evidence was of high quality.

**Interpretation:**

In this study, machine listening achieved excellent diagnostic accuracy, superior to the STOP-Bang (snoring, tiredness, observed apnea, BP, BMI, age, neck size, gender) questionnaire and comparable to common home sleep tests. Digital medicine should be further explored and externally validated for accessible and equitable OSA diagnosis.

**Clinical Trial Registration:**

PROSPERO database; No.: CRD42024534235; URL: https://www.crd.york.ac.uk/PROSPERO/).

**Take-Home Points**

**Study Question:** What is the diagnostic accuracy of machine listening for OSA, and how can it be optimized?

**Results:** Machine listening exhibited high diagnostic accuracy with a sensitivity of 90.3%, specificity of 86.7%, and a diagnostic OR of 60.8, with better performance when using higher audio sampling frequencies and non-contact microphones.

**Interpretation:** In this study, machine listening showed excellent diagnostic accuracy for OSA across the spectrum of severity, was comparable to home sleep tests, and should be further explored and validated for wider access to OSA diagnosis.

Among 1 billion patients worldwide with OSA,^1^ 90% remain undiagnosed.^2^ These patients experience disrupted sleep and intermittent oxygen desaturations,^3^ which places them at risk of serious complications, including heart disease,^4^ stroke,^5^ cognitive decline,^6^ depression, and cancer.^7^, ^8^, ^9^, ^10^, ^11^, ^12^, ^13^

The main barrier to diagnosis is the use of overnight polysomnography for testing, as it requires specialized equipment, skilled sleep technicians, and inpatient beds that are unavailable in primary care or developing countries. Screening tools such as the STOP-Bang (snoring, tiredness, observed apnea, BP, BMI, age, neck size, gender) questionnaire score are commonly used to estimate OSA risk in primary care and preoperative clinics.^14^ Although STOP-Bang is > 90% sensitive, it is only 30% specific, which results in numerous false-positive findings.^15^ Therefore, a pressing need exists for alternative accessible tests with good diagnostic accuracy for high-volume screening of patients with suspected OSA.

Recent advancements in artificial intelligence (AI) have enabled the use of breathing sound recordings with a smartphone to screen for OSA.^16^, ^17^, ^18^, ^19^, ^20^ AI can analyze various audio features such as breathing and snoring frequency, intensity, pitch, and duration to detect subtle acoustic patterns that differentiate normal and pathologic snoring. This approach offers a noninvasive, accessible, and potentially cost-effective method for the early detection of OSA.

Given the growing evidence base, it is timely to evaluate and optimize the diagnostic accuracy of this AI-based approach. In this Bayesian meta-analysis, the OSA diagnostic accuracy of AI models trained on breathing sound recordings was quantitatively pooled. Meta-regression was also used to identify factors associated with higher diagnostic accuracy.

## Study Design and Methods

This review is registered on PROSPERO (CRD42024534235) and is reported in accordance with the Preferred Reporting Items for Systematic Reviews and Meta-Analyses guidelines and the Meta-analysis Of Observational Studies in Epidemiology guidelines.^21^^,^^22^ The former checklist is included in e-Table 1.

### Search Strategy

PubMed, Embase, Scopus, Web of Science, and IEEE Xplore databases were searched from inception till July 20, 2024, using the following search strategy: (("sleep apnea" OR "sleep apnoea" OR "nocturnal hypoxia" OR "nocturnal hypoxaemia" OR "nocturnal hypoxemia" OR "sleep disordered breathing") AND ("artificial intelligence" OR "machine learning" OR "deep learning" OR "logistic regression" OR "support vector machine" OR "neural network" OR "classification tree" OR "regression tree" or "probability tree" OR "nearest neighbour" OR "nearest neighbor" OR "fuzzy logic" OR "naive bayes" OR "genetic algorithm" OR "multilayer perceptron" OR "random forest" OR "lasso regression" OR "kernel regression" OR “elastic net" OR "generative model" OR "generative adversarial network" OR "large language model") AND (diagnosis OR diagnose OR detect OR detection OR identify OR identification OR severity OR classify OR classification)). The full search strategy is available in the Supplemental Methods. Due to the extensive search strategy and large number of search results, no additional hand-searching was performed.

### Study Selection

Records were uploaded onto Rayyan,^23^ which is an online systematic review platform that enables authors to manually screen abstracts in a anonymized manner. At least 2 authors (E. Y. G., B. K. J. T., N. K. W. T., B. S. Y. Y.) independently selected potentially eligible studies based on title and abstract followed by full-text screening. Eligibility criteria were as follows.

Inclusion criteria:

1. Population: adults aged at least 18 years.
2. Intervention/exposure: diagnosis and classification of OSA using AI (eg, traditional regression techniques, machine learning) trained on breathing sound recordings.
3. Comparators: diagnosis and classification of OSA at the patient level using the apnea-hypopnea index (AHI) from overnight polysomnography or home sleep apnea tests.
4. Outcomes: Accuracy of AI in diagnosis and classification of OSA, assessed via a random split test set or k-fold cross-validation, and measured by sensitivity, specificity, positive predictive value, negative predictive value, and/or area under the curve (AUC).
5. Study type: observational studies (eg, cohort, cross-sectional).

Exclusion criteria:

1. Case reports, reviews, letters, conference abstracts, or other records not published as full-length articles in peer-reviewed journals.
2. Studies published in any language other than English that do not have an English translation.
3. Studies that were graded as having a high risk of bias across two or more domains.

### Data Extraction

Two authors extracted the following data from each article into a standardized extraction spreadsheet template: first author, year published, study design and setting, country, sample size (for the training, validation and test sets where available), percentage male, mean/median age, type of AI used, method of OSA diagnosis (eg, polysomnography or home sleep apnea tests), AHI cutoffs, OSA prevalence, and accuracy statistics (eg, sensitivity, specificity, accuracy, positive predictive value, negative predictive value, area under the curve [AUC]).

### Risk of Bias

The Quality Assessment of Diagnostic Accuracy Studies-2 (QUADAS-2) tool was used to evaluate the risk of bias and applicability of diagnostic accuracy studies.^24^ Two authors (B. K. J. T., E. Y. G.) independently graded study bias and applicability as low, high, or unclear, based on 4 key domains: patient selection, index test, reference standard, and flow and timing.

### Statistical Analysis

Binary diagnostic accuracy data were derived from the reported accuracy, sensitivity, specificity, and OSA prevalence. Studies that evaluated their model with a random split test set or k-fold cross-validation were pooled in a Bayesian bivariate random effects meta-analysis, using a noninformative prior. Pooled sensitivity and specificity were summarized by using summary receiver-operating characteristic (SROC) curves. Diagnostic ORs (DORs) and positive and negative likelihood ratios were also derived from the meta-analysis. Between-study heterogeneity was graphically visualized by using 95% prediction regions on SROC curves. An informative prior (where the lower bound was set as 50% sensitivity/specificity) was used as a sensitivity analysis for the overall meta-analysis. Random-effects Bayesian meta-regression analyses of continuous and categorical variables were performed. The potential impact of 4 different mechanisms of publication bias (data, sensitivity, specificity, or DOR-driven) with varying probabilities of unpublished studies (up to 60%) was evaluated via a sensitivity analysis in which the SROC curve, AUC, sensitivity, and specificity were re-estimated for each scenario in a Bayesian hierarchical framework.

All analyses were conducted following statistical guidance from the Cochrane Handbook and were performed using MetaBayesDTA (1.5.2) and DTAmetasa (0.9.1),^25^, ^26^, ^27^, ^28^, ^29^ built using R (R Foundation for Statistical Computing) and Stan (Stan Development Team).

## Results

The study selection process is summarized in e-Figure 1. From 6,254 nonduplicated records, 6,196 articles were excluded based on title and abstract screening, and 42 articles were excluded based on full-text screening. We included 16 studies (e-Table 2) with 41 AI models trained on 4,864 patients and tested on 2,370 patients.^16^, ^17^, ^18^, ^19^, ^20^^,^^30^, ^31^, ^32^, ^33^, ^34^, ^35^, ^36^, ^37^, ^38^, ^39^, ^40^

### Study Characteristics

#### Study Design, Setting, and Demographic Characteristics

All 16 studies were cross-sectional. Thirteen studies used data from patients recruited from hospital outpatient sleep clinics, and 2 studies used a convenience sample^17^^,^^20^; 1 did not specify the recruitment method.^19^ Eight, 4, and 2 studies were conducted in Asia,^16^, ^17^, ^18^^,^^32^, ^33^, ^34^^,^^36^^,^^39^ Europe,^19^^,^^20^^,^^35^^,^^37^ and Australasia, respectively^30^^,^^31^; 2 studies were conducted in both Asia and Europe.^38^^,^^40^ Mean age ranged from 40.5 to 52.1 years, and the percentage of male participants ranged from 49.5% to 84.1%. There were no studies with a high risk of bias based on QUADAS-2 (e-Table 3).

### Audio Recordings

Five studies used a non-contact smartphone to record breathing sounds at the bedside,^16^, ^17^, ^18^, ^19^, ^20^ 10 studies used a non-contact professional microphone,^30^, ^31^, ^32^, ^33^, ^34^^,^^36^, ^37^, ^38^, ^39^, ^40^ and 1 study used a professional microphone that was physically attached to the participant.^35^ Four studies recorded the audio in a home environment,^17^^,^^19^^,^^33^^,^^35^ and 12 studies recorded the audio in a controlled hospital environment.^16^^,^^18^^,^^20^^,^^30^, ^31^, ^32^^,^^34^^,^^36^, ^37^, ^38^, ^39^, ^40^

#### Reference Standard for OSA Diagnosis

Thirteen studies evaluated OSA using overnight polysomnography,^16^, ^17^, ^18^^,^^20^^,^^30^, ^31^, ^32^^,^^34^^,^^36^, ^37^, ^38^, ^39^, ^40^ while 3 studies used a home sleep apnea test.^19^^,^^33^^,^^35^ All studies defined OSA severity using the AHI. Among the 41 AI models, 10, 1, 16, and 14 models used an AHI cutoff of ≥ 5, ≥ 10, ≥ 15, and ≥ 30 events per hour, respectively, to define the presence of OSA. The prevalence of OSA ranged from 10.0% to 91.7%.

#### AI Models

Fourteen models used deep learning, and 27 models used traditional machine learning for feature extraction and selection. Eighteen models were evaluated with a train-test split, and 23 models were evaluated with a cross-validation technique (k-fold or leave-out-one).

### Meta-Analysis of Diagnostic Accuracy

#### Overall Accuracy Statistics

**Figure 1:**
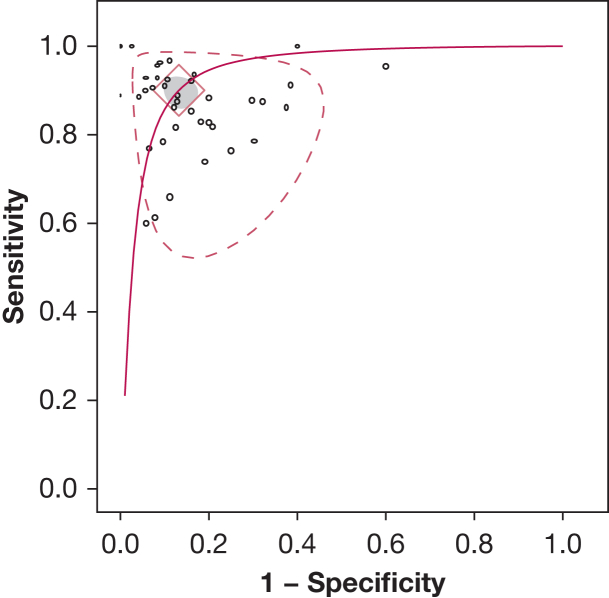
Summary receiver-operating characteristic plot for the overall OSA diagnostic accuracy of machine listening. The solid line represents the extrapolated summary receiver-operating characteristic curve. The diamond represents the summary receiver-operating point. Shaded/dashed regions represent the 95% credible/prediction intervals. Unshaded circles/ovals are centered around individual study means; their height/width are proportionate to study weights for sensitivity/specificity.

Compared with conventional diagnostic methods, the use of AI trained on breathing sound recordings achieved a pooled sensitivity of 90.3% (95% credible interval [95% CrI], 86.9%-93.1%) and a specificity of 86.7% (95% CrI, 83.1%-89.7%), with a DOR of 60.8 (95% CrI, 39.4-99.9), a positive likelihood ratio of 6.78 (95% CrI, 5.34-8.85), and a negative likelihood ratio of 0.113 (95% CrI, 0.079-0.152). The SROC curve is displayed in Figure 1, with additional details in e-Figure 2. A sensitivity analysis using an informative prior yielded identical results.

To put these findings in a diagnostic clinical context using the Bayes theorem,^41^ when the pretest probability (or baseline population prevalence of OSA) is approximately 15% (eg, Iceland, Indonesia, United Arab Emirates), 30% (eg, United States, United Kingdom, Samoa), or 60% (eg, Singapore, Switzerland, France),^1^ a positive test result would yield an average posttest probability of 54.5% (95% CrI, 48.5%-61.0%), 74.4% (95% CrI, 69.6%-79.1%), or 91.0% (95% CrI, 88.9%-93.0%), whereas a negative test result would yield an average posttest probability of 2.0% (95% CrI, 1.4%-2.6%), 4.6% (95% CrI, 3.3%-6.1%), or 14.5% (95% CrI, 10.6%-18.6%), respectively. In clinical situations in which the patient has a pretest probability of OSA that is already higher than the baseline regional OSA prevalence (eg, if the patient has symptoms or signs of OSA), the posttest probability would be even higher.

#### Meta-Regression of AHI Cutoffs

**Figure 2:**
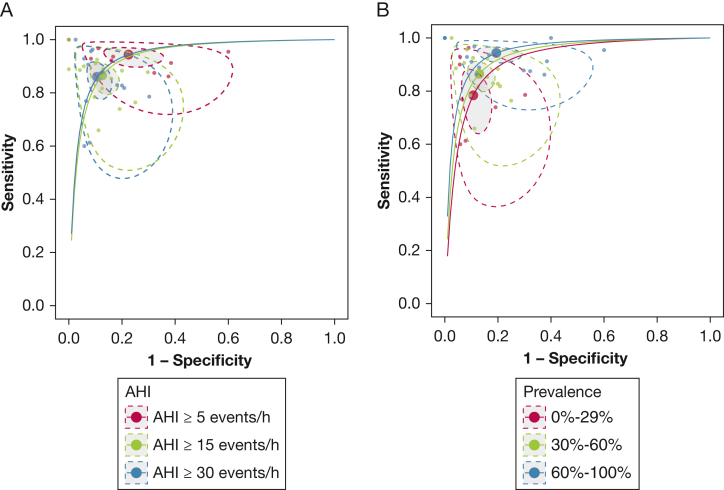
Summary receiver-operating characteristic plot for Bayesian meta-regression stratified by AHI cutoff (A) and OSA prevalence (B). Solid lines represent the extrapolated summary receiver-operating characteristic curves. Large circles represent the summary operating points. The shaded areas represent the 95% credible regions, and the dotted lines represent the 95% prediction region. Small circles represent individual study estimates. AHI = apnea-hypopnea index.

At the clinically relevant AHI cutoffs of ≥ 5, ≥ 15, and ≥ 30 events per hour, the sensitivities were 94.3% (95% CrI, 90.3%-96.8%), 86.3% (95% CrI, 80.1%-90.9%), and 86.3% (95% CrI, 79.2%-91.1%), and the specificities were 78.5% (95% CrI, 68.0%-86.9%), 87.3% (95% CrI, 81.8%-91.3%), and 89.5% (95% CrI, 84.8%-93.3%), respectively. Other statistics are summarized in Table 1. Visual comparison of the 3 SROC curves for each AHI cutoff suggested that performance was essentially identical across all 3 AHI cutoffs, as the curves were almost completely overlapping (Fig 2A) despite minor differences in the summary operating point.

**Table 1:** Summary of Diagnostic Test Accuracy Statistics From Bayesian Meta-Analysis at Clinically Relevant Thresholds

Subgroup	Posterior Median (95% Posterior Interval)					
	Sensitivity	Specificity	FPR	DOR	LR+	LR–
Overall	90.3 (86.9-93.1)	86.7 (83.1-89.7)	13.3 (10.3-16.9)	60.7 (39.4-99.9)	6.77 (5.34-8.85)	0.113 (0.079-0.152)
AHI ≥ 5 events/h	94.3 (90.3-96.8)	78.5 (68.0-86.9)	21.5 (13.1-32)	60.6 (24.8-161)	4.38 (2.92-7.20)	0.073 (0.039-0.129)
AHI ≥ 15 events/h	86.3 (80.1-90.9)	87.3 (81.8-91.3)	12.7 (8.7-18.2)	43.9 (20.8-87.2)	6.8 (4.59-10.1)	0.156 (0.103-0.238)
AHI ≥ 30 events/h	86.3 (79.2-91.1)	89.5 (84.8-93.3)	10.5 (6.7-15.2)	53.9 (25.9-117)	8.17 (5.53-12.9)	0.154 (0.099-0.236)

AHI = apnea-hypopnea index; DOR = diagnostic OR; FPR = false positive rate (1 – specificity); LR+ = positive likelihood ratio; LR– = negative likelihood ratio.

#### Meta-Regression of Demographic Characteristics

Greater OSA prevalence (Fig 2B), stratified as low (0%-29%), medium (30%-60%), and high prevalence (60%-100%), was associated with a significantly higher sensitivity (difference, 15.7 [95% CrI, 6.2%-29.9%], high vs low prevalence; 7.9% [95% CrI, 2.4%-13.8%], high vs medium prevalence) but not specificity. Conversely, average age and the percentage of male participants were not associated with sensitivity or specificity (e-Fig 3).

#### Meta-Regression of Audio Recording Characteristics

**Figure 3:**
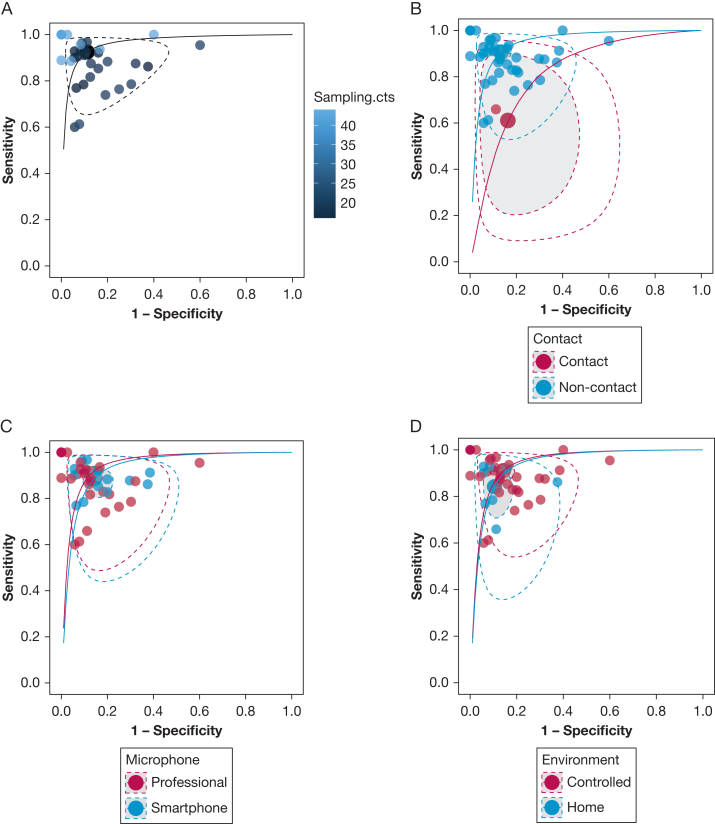
Summary receiver-operating characteristic (SROC) plot for Bayesian meta-regression stratified by audio sampling frequency (A), microphone position (B), microphone type (C), and environment (D). Solid lines represent the extrapolated SROC curves. Large circles represent the summary operating points. The shaded areas represent the 95% credible regions, and the dotted lines represent the 95% prediction region. Small circles represent individual study estimates.

Sensitivity but not specificity was significantly higher among AI models that used a higher sampling frequency for their microphone (coefficient, 0.078; 95% CrI, 0.041-0.120) (Fig 3A) and non-contact rather than contact microphones (difference in sensitivity, 30.1%; 95% CrI, 1.7%-67.5%) (Fig 3B). The type of microphone (professional/smartphone) (Fig 3C) and recording environment (home/controlled) (Fig 3D) were not associated with sensitivity/specificity, suggesting that AI models trained on smartphone recordings in a home environment were as equally accurate as those trained on professional microphone recordings in controlled environments.

#### Meta-Regression of AI Characteristics

The type of feature engineering (deep learning/domain expert) and classifier (deep learning/traditional machine learning) were not associated with sensitivity/specificity (e-Fig 4). Studies that evaluated models using a train-test split had higher sensitivity (difference, 6.4%; 95% CrI, 0.8%-13.1%) but similar specificity as studies that used cross-validation.

#### Publication Bias

**Figure 4:**
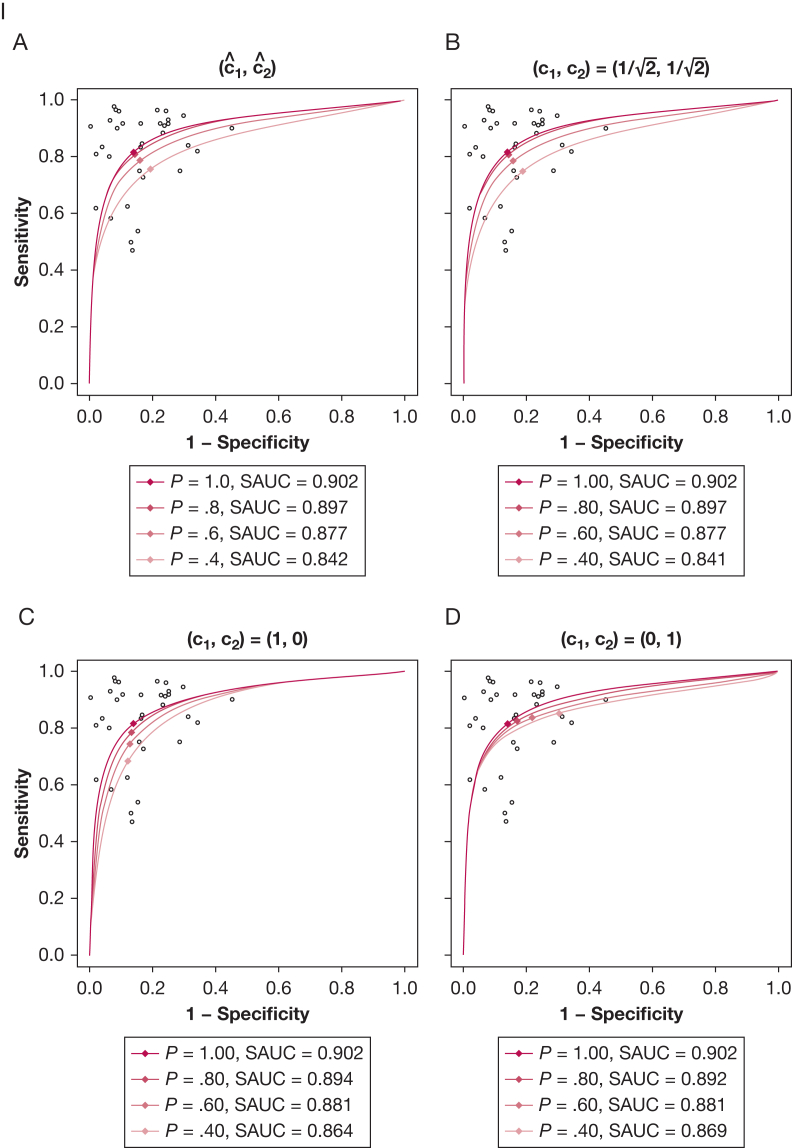

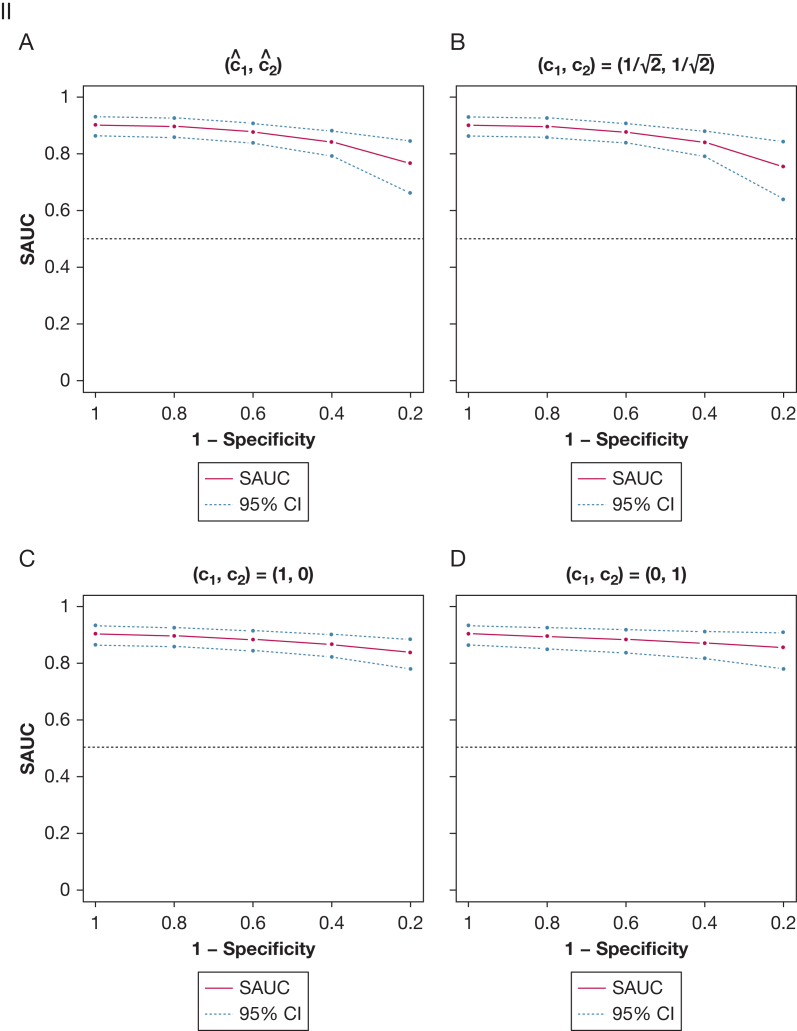
Effect of varying scenarios of publication bias on the summary receiver-operating characteristic (SROC) curve (I) and area under the curve (AUC) (II). The potential impact of 4 different mechanisms of publication bias is shown: data-driven (A), sensitivity-driven (B), specificity-driven (C), and diagnostic OR-driven (D) with varying probabilities of unpublished studies (0%, 20%, 40%, and 60%). In (I), solid lines and diamonds represent the SROC curves and summary operating points. Blue and red dots represent the mean and 95% CIs of the AUC for each probability of unpublished studies (x-axis). SAUC = area under the SROC curve.

Sensitivity analyses on the SROC curve and AUC suggested no clinically significant publication bias. When considering 4 different mechanisms of publication bias (data, sensitivity, specificity, or DOR-driven), with varying probabilities of unpublished studies (up to 60%), the SROC curve and AUC were almost constant (Fig 4). This suggests that even if most studies remained unpublished, the conclusions of this meta-analysis would not have changed.

### Quality of Evidence

e-Table 4 summarizes the quality of evidence at the outcome level. The overall quality of evidence was high. There was clear evidence of a sensitivity-specificity relationship.

## Discussion

This study systematically reviewed the diagnostic accuracy of AI models trained on breathing sound recordings for the diagnosis of OSA. Among 41 AI models, the pooled overall sensitivity and specificity were approximately 90% and 87%, respectively. Meta-regression revealed that non-contact microphones, higher audio sampling frequency, higher OSA prevalence, and train-test splits were associated with higher sensitivity. Performance was overall similar across various clinically relevant AHI cutoffs for OSA severity. There was no evidence to suggest that the findings were affected by publication bias.

This study is currently the most comprehensive evidence synthesis of AI models utilizing breathing sound recordings for OSA diagnosis. Previous research has primarily focused on clinical data, polysomnographic markers,^42^ facial photographs,^43^ or wearable devices,^44^ or did not perform a quantitative synthesis.^45^ The current study highlights the promising utility of acoustic features in AI-driven diagnostics, which is evident from the high sensitivity and specificity observed in this meta-analysis. The meta-analysis showed that AI models trained on breathing sound recordings had a pooled sensitivity and specificity of 94.3% and 78.5% at AHI ≥ 5 events per hour, and 86.3% and 87.3% at AHI ≥ 15 events per hour. These findings are far superior to the STOP-Bang risk score (AHI ≥ 5 events per hour, 91.4% sensitivity and 33.5% specificity; AHI ≥ 15 events per hour, 94.4% sensitivity and 27.8% specificity)^15^ and at least comparable to commonly used home sleep apnea tests such as WatchPAT (Itamar Medical Ltd) (AHI ≥ 5 events per hour, 94.1% and 43.5%; AHI ≥ 10 events per hour, 92.2% and 72.4%).^46^ Furthermore, AI models trained on acoustic features have the key advantage of not requiring any specialized devices or wearables, apart from the ubiquitous smartphone. Therefore, this approach is a potentially scalable, noninvasive, and cost-effective alternative to conventional diagnostic methods.

The meta-regression findings suggest several plausible explanations for the observed inter-model variations in diagnostic accuracy. The higher sensitivity associated with non-contact microphones may be explained by less interfering noise from rubbing against the participant's body or clothes during sleep. Using a higher audio sampling frequency may improve the temporal resolution of breathing sound signals, which may account for the higher sensitivity observed. The AI models seem to have similar accuracy regardless of the age or sex of participants, which increases their generalizability. In addition, smartphone recordings done in a home setting seem to be as similarly effective as professional recordings done in the controlled sleep laboratory, which highlights the real-world applicability of this approach.

Interestingly, deep learning did not perform significantly better than traditional machine learning in this meta-analysis. This is likely because the current sample size is simply too small, with a training set of 4,864 patients and a test set of 2,370 patients. As data set size increases, deep learning often continues to improve in accuracy, long after traditional machine learning plateaus.^47^ This can be explained by deep learning’s multiple hidden layers of artificial neurons modeled after the human brain, which allows better recognition of complex, subtle relationships than traditional machine learning.^48^ In the field of audio signal processing (feature engineering), deep learning models similarly outperform traditional techniques such as Gaussian mixture models and hidden Markov models when sufficient data are available.^49^ It is thus important for future studies to enhance model robustness through larger, more diverse training data sets.

Because the current meta-analysis included studies using only audio recordings without clinical variables, there is further room for improved accuracy by integrating multimodal data. This may range from clinical data such as questionnaires to the use of various polysomnography signals, such as airflow, pulse oximetry, ECG, or EEG. These signals have been extensively studied for training AI models, with reported diagnostic accuracy ranging from 70% to 90% sensitivity and specificity, depending on the study and AI model, with deep learning approaches generally yielding higher accuracy.^45^ Although a comprehensive review of these alternative AI approaches is beyond the scope of the current work, models using these signals could complement breathing sound recordings by capturing essential physiologic features such as oxygen desaturation and respiratory effort. Therefore, further comparative studies and multimodal approaches are necessary to explore potential synergies and clarify the optimal use cases for each modality.

A major strength of the current study is the rigorous selection and evaluation of included studies, ensuring a high quality of evidence. The use of QUADAS-2 for bias assessment and extensive sensitivity analyses to check for publication bias further strengthen the reliability of the findings. However, some limitations must be addressed. The heterogeneity in recording environments, types of microphones, and AI models could introduce variability in the results. The reliance on breathing sounds alone may not capture all relevant features of OSA such as oxygen desaturation and the hypoxic burden, which is mainly responsible for the morbidity associated with OSA.^50^ AI trained on acoustic features may also underdiagnose patients with OSA who do not snore. There was a large variation in the prevalence of OSA, which partly reflects the varying AHI cutoffs used but may also represent sample or population variability in OSA prevalence. Within-study clustering (due to multiple AI models from the same study, mostly examining different AHI cutoffs) could not be explicitly modeled in a hierarchical manner using the current statistical package. This was somewhat mitigated by the reporting of subgroup meta-analyses according to AHI cutoff, in which almost every study was represented just once. Importantly, the generalizability of findings to diverse populations and settings remains to be externally validated, as only one of the included studies had performed external validation in a completely new, large cohort.^17^ Furthermore, because 13 of 16 studies recruited patients from hospital outpatient sleep clinics, these AI models have not been validated in lower prevalence clinical contexts. Therefore, future prospective cohorts for external validation, particularly in lower prevalence settings, are still required prior to the widespread implementation of this approach.

## Interpretation

High-quality evidence suggests that AI models trained only on breathing sound recordings can achieve an excellent OSA diagnostic accuracy of 87% to 90%. Digital medicine and AI should be further explored to improve the accessibility of OSA diagnosis, especially in primary care and resource-limited settings.

## Funding/Support

The authors have reported to *CHEST* that no funding was received for this study.

## Financial/Nonfinancial Disclosures

None declared.

## Supplementary Data

e-Online Data
