# Supplementary material for: Machine Listening for OSA Diagnosis: A Bayesian Meta-Analysis
Source: Chest. 2025 Apr 11;168(2):520–30. doi: 10.1016/j.chest.2025.04.006 (PMC12405919; doi:10.1016/j.chest.2025.04.006)
Supplement: e-Online Data [file mmc1.docx]

[SUPPLEMENTAL METHODS 2](#_Toc194005865)

[Search Strategy 2](#_Toc194005866)

[SUPPLEMENTAL MATERIAL 4](#_Toc194005867)

[e-Figure 1 4](#_Toc194005868)

[e-Figure 2 5](#_Toc194005869)

[e-Figure 3 6](#_Toc194005870)

[e-Figure 4 8](#_Toc194005871)

[SUPPLEMENTAL TABLES 11](#_Toc194005872)

[e-Table 1 11](#_Toc194005873)

[e-Table 2 13](#_Toc194005874)

[e-Table 3 16](#_Toc194005875)

[e-Table 4 17](#_Toc194005876)

# SUPPLEMENTAL METHODS

## Search Strategy

Overall search strategy:

Free text search strategy: (("sleep apnea" OR "sleep apnoea" OR "nocturnal hypoxia" OR "nocturnal hypoxaemia" OR "nocturnal hypoxemia" OR "sleep disordered breathing") AND ("artificial intelligence" OR "machine learning" OR "deep learning" OR "logistic regression" OR "support vector machine" OR "neural network" OR "classification tree" OR "regression tree" or "probability tree" OR "nearest neighbour" OR "nearest neighbor" OR "fuzzy logic" OR "naive bayes" OR "genetic algorithm" OR "multilayer perceptron" OR "random forest" OR "lasso regression" OR "kernel regression" OR “elastic net" OR "generative model" OR "generative adversarial network" OR "large language model") AND (diagnosis OR diagnose OR detect OR detection OR identify OR identification OR severity OR classify OR classification))

Pubmed search strategy:

("sleep apnea"[All Fields] OR "sleep apnoea"[All Fields] OR "nocturnal hypoxia"[All Fields] OR "nocturnal hypoxaemia"[All Fields] OR "nocturnal hypoxemia"[All Fields] OR "sleep disordered breathing"[All Fields]) AND ("artificial intelligence"[All Fields] OR "machine learning"[All Fields] OR "deep learning"[All Fields] OR "logistic regression"[All Fields] OR "support vector machine"[All Fields] OR "neural network"[All Fields] OR "classification tree"[All Fields] OR "regression tree"[All Fields] OR "probability tree"[All Fields] OR "nearest neighbour"[All Fields] OR "nearest neighbor"[All Fields] OR "fuzzy logic"[All Fields] OR "naive bayes"[All Fields] OR "genetic algorithm"[All Fields] OR "multilayer perceptron"[All Fields] OR "random forest"[All Fields] OR "lasso regression"[All Fields] OR "kernel regression"[All Fields] OR "elastic net"[All Fields] OR "generative model"[All Fields] OR "generative adversarial network"[All Fields] OR "large language model"[All Fields]) AND ("diagnosable"[All Fields] OR "diagnosi"[All Fields] OR "diagnosis"[MeSH Terms] OR "diagnosis"[All Fields] OR "diagnose"[All Fields] OR "diagnosed"[All Fields] OR "diagnoses"[All Fields] OR "diagnosing"[All Fields] OR "diagnosis"[MeSH Subheading] OR ("diagnosable"[All Fields] OR "diagnosi"[All Fields] OR "diagnosis"[MeSH Terms] OR "diagnosis"[All Fields] OR "diagnose"[All Fields] OR "diagnosed"[All Fields] OR "diagnoses"[All Fields] OR "diagnosing"[All Fields] OR "diagnosis"[MeSH Subheading]) OR ("detect"[All Fields] OR "detectabilities"[All Fields] OR "detectability"[All Fields] OR "detectable"[All Fields] OR "detectables"[All Fields] OR "detectably"[All Fields] OR "detected"[All Fields] OR "detectible"[All Fields] OR "detecting"[All Fields] OR "detection"[All Fields] OR "detections"[All Fields] OR "detects"[All Fields]) OR ("detect"[All Fields] OR "detectabilities"[All Fields] OR "detectability"[All Fields] OR "detectable"[All Fields] OR "detectables"[All Fields] OR "detectably"[All Fields] OR "detected"[All Fields] OR "detectible"[All Fields] OR "detecting"[All Fields] OR "detection"[All Fields] OR "detections"[All Fields] OR "detects"[All Fields]) OR ("identifiable"[All Fields] OR "identifiably"[All Fields] OR "identifie"[All Fields] OR "identified"[All Fields] OR "identifier"[All Fields] OR "identifiers"[All Fields] OR "identifies"[All Fields] OR "identify"[All Fields] OR "identifying"[All Fields]) OR ("identifed"[All Fields] OR "identification, psychological"[MeSH Terms] OR ("identification"[All Fields] AND "psychological"[All Fields]) OR "psychological identification"[All Fields] OR "identification"[All Fields] OR "identifications"[All Fields]) OR ("sever"[All Fields] OR "severe"[All Fields] OR "severed"[All Fields] OR "severely"[All Fields] OR "severer"[All Fields] OR "severes"[All Fields] OR "severing"[All Fields] OR "severities"[All Fields] OR "severity"[All Fields] OR "severs"[All Fields]) OR ("classifiable"[All Fields] OR "classification"[MeSH Terms] OR "classification"[All Fields] OR "classified"[All Fields] OR "classify"[All Fields] OR "classifying"[All Fields] OR "classifier"[All Fields] OR "classifier s"[All Fields] OR "classifiers"[All Fields] OR "classifies"[All Fields]) OR ("classification"[MeSH Terms] OR "classification"[All Fields] OR "classifications"[All Fields] OR "classification"[MeSH Subheading] OR "classification s"[All Fields] OR "classificator"[All Fields] OR "classificators"[All Fields]))

Embase search strategy:

('sleep apnea'/exp OR 'sleep apnea' OR 'sleep apnoea'/exp OR 'sleep apnoea' OR 'nocturnal hypoxia'/exp OR 'nocturnal hypoxia' OR 'nocturnal hypoxaemia' OR 'nocturnal hypoxemia'/exp OR 'nocturnal hypoxemia' OR 'sleep disordered breathing'/exp OR 'sleep disordered breathing') AND ('artificial intelligence'/exp OR 'artificial intelligence' OR 'machine learning'/exp OR 'machine learning' OR 'deep learning'/exp OR 'deep learning' OR 'logistic regression'/exp OR 'logistic regression' OR 'support vector machine'/exp OR 'support vector machine' OR 'neural network'/exp OR 'neural network' OR 'classification tree'/exp OR 'classification tree' OR 'regression tree'/exp OR 'regression tree' OR 'probability tree' OR 'nearest neighbour' OR 'nearest neighbor' OR 'fuzzy logic'/exp OR 'fuzzy logic' OR 'naive bayes'/exp OR 'naive bayes' OR 'genetic algorithm'/exp OR 'genetic algorithm' OR 'multilayer perceptron'/exp OR 'multilayer perceptron' OR 'random forest'/exp OR 'random forest' OR 'lasso regression'/exp OR 'lasso regression' OR 'kernel regression'/exp OR 'kernel regression' OR 'elastic net'/exp OR 'elastic net' OR 'generative model'/exp OR 'generative model' OR 'generative adversarial network'/exp OR 'generative adversarial network' OR 'large language model') AND ('diagnosis'/exp OR diagnosis OR diagnose OR detect OR 'detection'/exp OR detection OR identify OR 'identification'/exp OR identification OR 'severity'/exp OR severity OR classify OR 'classification'/exp OR classification) NOT [medline]/lim AND ([article]/lim OR [article in press]/lim) AND [english]/lim

Scopus search strategy:

( "sleep apnea" OR "sleep apnoea" OR "nocturnal hypoxia" OR "nocturnal hypoxaemia" OR "nocturnal hypoxemia" OR "sleep disordered breathing" ) AND ( "artificial intelligence" OR "machine learning" OR "deep learning" OR "logistic regression" OR "support vector machine" OR "neural network" OR "classification tree" OR "regression tree" OR "probability tree" OR "nearest neighbour" OR "nearest neighbor" OR "fuzzy logic" OR "naive bayes" OR "genetic algorithm" OR "multilayer perceptron" OR "random forest" OR "lasso regression" OR "kernel regression" OR "elastic net" OR "generative model" OR "generative adversarial network" OR "large language model" ) AND ( diagnosis OR diagnose OR detect OR detection OR identify OR identification OR severity OR classify OR classification ) AND NOT INDEX ( medline ) AND ( LIMIT-TO ( DOCTYPE , "ar" ) ) AND ( LIMIT-TO ( LANGUAGE , "English" ) ) AND ( LIMIT-TO ( EXACTKEYWORD , "Human" ) )

Scopus search strategy:

( "sleep apnea" OR "sleep apnoea" OR "nocturnal hypoxia" OR "nocturnal hypoxaemia" OR "nocturnal hypoxemia" OR "sleep disordered breathing" ) AND ( "artificial intelligence" OR "machine learning" OR "deep learning" OR "logistic regression" OR "support vector machine" OR "neural network" OR "classification tree" OR "regression tree" OR "probability tree" OR "nearest neighbour" OR "nearest neighbor" OR "fuzzy logic" OR "naive bayes" OR "genetic algorithm" OR "multilayer perceptron" OR "random forest" OR "lasso regression" OR "kernel regression" OR "elastic net" OR "generative model" OR "generative adversarial network" OR "large language model" ) AND ( diagnosis OR diagnose OR detect OR detection OR identify OR identification OR severity OR classify OR classification ) AND NOT INDEX ( medline ) AND ( LIMIT-TO ( DOCTYPE , "ar" ) ) AND ( LIMIT-TO ( LANGUAGE , "English" ) )

Web of science search strategy:

("sleep apnea" OR "sleep apnoea" OR "nocturnal hypoxia" OR "nocturnal hypoxaemia" OR "nocturnal hypoxemia" OR "sleep disordered breathing") AND ("artificial intelligence" OR "machine learning" OR "deep learning" OR "logistic regression" OR "support vector machine" OR "neural network" OR "classification tree" OR "regression tree" or "probability tree" OR "nearest neighbour" OR "nearest neighbor" OR "fuzzy logic" OR "naive bayes" OR "genetic algorithm" OR "multilayer perceptron" OR "random forest" OR "lasso regression" OR "kernel regression" OR “elastic net" OR "generative model" OR "generative adversarial network" OR "large language model") AND (diagnosis OR diagnose OR detect OR detection OR identify OR identification OR severity OR classify OR classification)

With the following limits applied: article, English language

IEEE Xplore search strategy:

("sleep apnea" OR "nocturnal hypoxemia" OR "sleep disordered breathing") AND ("artificial intelligence" OR "machine learning" OR "deep learning" OR "logistic regression" OR "support vector machine" OR "neural network" OR "classification tree" OR "regression tree" or "probability tree" OR "nearest neighbor" OR "random forest" OR "generative model" OR "generative adversarial network" OR "large language model") AND (diagnos* OR detect* OR identif* OR severity OR classif*)

# SUPPLEMENTAL MATERIAL

e-Figure 1**:** **Preferred Reporting Items for Systematic reviews and Meta-Analyses (PRISMA) flow diagram to summarize the study selection process.**

e-Figure 2**: Summary receiver operating characteristic (SROC) plot for the overall OSA diagnostic accuracy of machine listening, with 95% confidence intervals for sensitivity (top), specificity (middle) and both (bottom) displayed for individual models.**

e-Figure 3**: Accuracy versus covariate plot for Bayesian meta-regression of (A) average age and (B) percentage male.**

**e-Figure 3A:**

**e-Figure 3B:**

e-Figure 4**: Accuracy versus covariate plot for Bayesian meta-regression of (A) feature engineering (deep learning/domain expert), (B) classifier (deep learning/traditional machine learning [ML]), (C) model evaluation (random split/cross-validation).**

**e-Figure 4A:**

**e-Figure 4B:**

**e-Figure 4C:**

# SUPPLEMENTAL TABLES

e-Table 1**: Preferred Reporting Items for Systematic reviews and Meta-Analyses (PRISMA) checklist.**

| **Item No.** | **Recommendation** | **Reported on Page No** |
| --- | --- | --- |
| Reporting of background should include | | |
| 1 | Problem definition | 3 |
| 2 | Hypothesis statement | - |
| 3 | Description of study outcome(s) | 4 |
| 4 | Type of exposure or intervention used | 4 |
| 5 | Type of study designs used | 4 |
| 6 | Study population | 4 |
| Reporting of search strategy should include | | |
| 7 | Qualifications of searchers (eg, librarians and investigators) | Title page |
| 8 | Search strategy, including time period included in the synthesis and key words | 4, Supplemental Methods |
| 9 | Effort to include all available studies, including contact with authors | 4 |
| 10 | Databases and registries searched | 4 |
| 11 | Search software used, name and version, including special features used (eg, explosion) | Supplemental Methods |
| 12 | Use of hand searching (eg, reference lists of obtained articles) | 4 |
| 13 | List of citations located and those excluded, including justification | 5, Fig 1 |
| 14 | Method of addressing articles published in languages other than English | 4 |
| 15 | Method of handling abstracts and unpublished studies | 4 |
| 16 | Description of any contact with authors | No contact required |
| Reporting of methods should include | | |
| 17 | Description of relevance or appropriateness of studies assembled for assessing the hypothesis to be tested | 4 |
| 18 | Rationale for the selection and coding of data (eg, sound clinical principles or convenience) | 4-5 |
| 19 | Documentation of how data were classified and coded (eg, multiple raters, blinding and interrater reliability) | 4-5 |
| 20 | Assessment of confounding (eg, comparability of cases and controls in studies where appropriate) | 5 |
| 21 | Assessment of study quality, including blinding of quality assessors, stratification or regression on possible predictors of study results | 5 |
| 22 | Assessment of heterogeneity | 5 |
| 23 | Description of statistical methods (eg, complete description of fixed or random effects models, justification of whether the chosen models account for predictors of study results, dose-response models, or cumulative meta-analysis) in sufficient detail to be replicated | 5 |
| 24 | Provision of appropriate tables and graphics | Tables & Figures |
| Reporting of results should include | | |
| 25 | Graphic summarizing individual study estimates and overall estimate | Fig S1 |
| 26 | Table giving descriptive information for each study included | Table S2 |
| 27 | Results of sensitivity testing (eg, subgroup analysis) | 6-7 |
| 28 | Indication of statistical uncertainty of findings | Always reported |
| Reporting of discussion should include | | |
| 29 | Quantitative assessment of bias (eg, publication bias) | 7 |
| 30 | Justification for exclusion (eg, exclusion of non-English language citations) | Fig S1 |
| 31 | Assessment of quality of included studies | Table S3 |
| Reporting of conclusions should include | | |
| 32 | Consideration of alternative explanations for observed results | 8-9 |
| 33 | Generalization of the conclusions (ie, appropriate for the data presented and within the domain of the literature review) | 8-9 |
| 34 | Guidelines for future research | 9 |
| 35 | Disclosure of funding source | 9 |

*From*: Stroup DF, Berlin JA, Morton SC, et al, for the Meta-analysis Of Observational Studies in Epidemiology (MOOSE) Group. Meta-analysis of Observational Studies in Epidemiology. A Proposal for Reporting. *JAMA*. 2000;283(15):2008-2012. doi: 10.1001/jama.283.15.2008.

e-Table 2**: Summary of included studies.**

| **First Author & Year DOI** | **Reference Standard** | **OSA Prevalence for each AHI** | **Country** | **Sample Size Average Age % Male** | **Microphone**  **Sampling Frequency (Hz)**  **Contact / Non-contact**  **Environment** | **Feature Engineering Classification Model Evaluation** |
| --- | --- | --- | --- | --- | --- | --- |
| Akhter 2018 10.5664/jcsm.7168 | PSG AHI >=15, 30 | 64, 47.7 | Australia | 91 61.5 50.2 | Professional NR Non-contact Controlled | Deep Learning Logistic Regression Cross-validation |
| Cho 2022 10.1001/jamaoto.2022.0244 | PSG AHI >=5, 15, 30 | 89.8, 70.9, 45.2 | South Korea | 423 84.1 48.1 | Smartphone NR Non-contact Controlled | Domain expert Random Forest Random split |
| de Silva 2012 10.1109/EMBC.2012.6347447 | PSG AHI >=15 | 45.7, 37.1 (female), 76.5, 54.9 (male) | Australia | 87 58.6 51.6 | Professional 20 Non-contact Controlled | Domain expert Logistic Regression Cross-validation |
| Ding 2024 10.1088/1361-6579/ad4953 | PSG AHI >=5, 10 | 91.7, 85 | China, Greece | 120 79.2 46.8 | Professional 44 Non-contact Controlled | Deep Learning K-Nearest-Neighbors Random split |
| Han 2024 10.1001/jamaoto.2023.3490 | PSG AHI >=5, 15, 30 | 50.5, 29.7, 13.9 | South Korea | 1416 49.5 48.3 | Smartphone 16 Non-contact Home | Deep Learning Deep Learning Random split |
| Kim 2019 10.21053/ceo.2018.00388 | PSG AHI >=5, 15, 30 | 75.9, 51.7, 25.9 | South Korea | 116 67.2 50.4 | Professional 20 Non-contact Controlled | Domain expert Logistic Regression Cross-validation |
| Le 2023 10.2196/44818 | PSG AHI >=5, 15, 30 | 82, 50, 18 | South Korea | 1315 72.24 52.13 | Smartphone 20 Non-contact Controlled | Deep Learning Linear Regression Random split |
| Li 2023 10.1016/j.bspc.2023.104966 | HSAT AHI >=5 | 70 | China | 124 74.2 45.5 | Professional 16 Non-contact Home | Deep Learning Deep Learning Random split |
| Luo 2020 10.1088/1361-6579/ab9e7b | PSG AHI >=15, 30 | 16.7, 10 | China | 132 82.6 40.5 | Professional 22 Non-contact Controlled | Deep Learning Logistic Regression Cross-validation |
| Roebuck 2015 10.3389/fbioe.2015.00114 | HSAT AHI >=15 | 52 | United Kingdom | 858 68.3 49.3 | Professional NR Contact Home | Domain expert Random Forest Cross-validation |
| Romero 2022 10.1109/JBHI.2022.3154719 | HSAT AHI >=5, 15, 30 | 91.7, 49, 24.8 | United Kingdom | 103 65 45 | Smartphone 16 Non-contact Home | Domain expert Deep Learning Cross-validation |
| Song 2023 10.1016/j.amjoto.2023.103964 | PSG AHI >=15, 30 | NR | China, Greece | 40 NR 49.3 | Professional 44 Non-contact Controlled | Domain expert Deep Learning Random split |
| Tiron 2020 10.21037/jtd-20-804 | PSG AHI >=15 | 50 | Germany | 248 54.1 50.5 | Smartphone 22 Non-contact Controlled | Domain expert Deep Learning Random split |
| Wang 2022 10.2147/NSS.S373367 | PSG AHI >=5, 15, 30 | 79.7, 59.3, 39 | China | 194 75.8 40.9 | Professional 44 Non-contact Controlled | Domain expert Deep Learning Random split |
| Xie 2023 10.1016/j.bspc.2023.104942 | PSG AHI >=5, 15, 30 | 76.7, 41.9, 18 | Netherlands | 172 63.4 50.3 | Professional 23 Non-contact Controlled | Domain expert Xtreme Gradient Boosting Cross-validation |
| Ye 2024 10.1007/s13246-023-01345-1 | PSG AHI >=5, 15, 30 | 90.4, 78.7, 58.5 | China | 94 NR 45.1 | Professional 44 Non-contact Controlled | Domain expert Support Vector Machine Cross-validation |

Abbreviations: PSG, polysomnography; HSAT, home sleep apnea test; AHI, apnea-hypopnea index.

e-Table 3**: Evaluation of risk of bias using the Quality Assessment of Diagnostic Accuracy Studies-2 (QUADAS-2) tool for diagnostic accuracy.**

| **Study** | **QUADAS-2 risk of bias** | | | | **QUADAS-2 concerns regarding applicability** | | | **Risk of bias** |
| --- | --- | --- | --- | --- | --- | --- | --- | --- |
|  | **Patient Selection** | **Index Test** | **Reference Standard** | **Flow & Timing** | **Patient Selection** | **Index Test** | **Reference Standard** |  |
| Akhter 2018 | Low | Unclear | Low | Low | Low | Low | Low | Unclear |
| Cho 2022 | Low | Low | Low | Low | Low | Low | Low | Low |
| de Silva 2012 | Unclear | Unclear | Low | Low | Low | Low | Low | Unclear |
| Ding 2024 | Unclear | Low | Low | Low | Low | Low | Low | Unclear |
| Han 2024 | Unclear | Low | Low | Low | Low | Low | Low | Low |
| Kim 2019 | Low | Unclear | Low | Low | Low | Low | Low | Unclear |
| Le 2023 | Low | Low | Low | Low | Low | Low | Low | Low |
| Li 2023 | Low | Low | Unclear | Low | Low | Low | Low | Unclear |
| Luo 2020 | Low | Unclear | Low | Low | Low | Low | Low | Unclear |
| Roebuck 2015 | Low | Unclear | Unclear | Low | Low | Low | Low | Unclear |
| Romero 2022 | Unclear | Unclear | Unclear | Low | Low | Low | Low | Unclear |
| Song 2023 | Low | Low | Low | Low | Low | Low | Low | Low |
| Tiron 2020 | Unclear | Low | Low | Low | Low | Low | Low | Unclear |
| Wang 2022 | Low | Low | Low | Low | Low | Low | Low | Low |
| Xie 2023 | Low | Unclear | Low | Low | Low | Low | Low | Unclear |
| Ye 2024 | Unclear | Unclear | Low | Low | Low | Low | Low | Unclear |

e-Table 4**: Evaluation of quality of pooled evidence using the Grading of Recommendations Assessment, Development and Evaluation (GRADE) framework**

**Question**: Should artificial intelligence trained on snoring audio recordings be used to screen for OSA in adults who snore?

| \| Sensitivity \| 0.90 (95% CI: 0.87 to 0.93) \| \| --- \| --- \| \| Specificity \| 0.87 (95% CI: 0.83 to 0.90) \| |  | \| Prevalences \| 15% \| 30% \| 60% \| \| --- \| --- \| --- \| --- \| |  |
| --- | --- | --- | --- | --- | --- | --- | --- | --- | --- | --- | --- |

| Outcome | № of studies (№ of patients) | Study design | Factors that may decrease certainty of evidence | | | | | Effect per 1,000 patients tested | | | Test accuracy CoE |
| --- | --- | --- | --- | --- | --- | --- | --- | --- | --- | --- | --- |
|  |  |  | Risk of bias | Indirectness | Inconsistency | Imprecision | Publication bias | pre-test probability of 15% | pre-test probability of 30% | pre-test probability of 60% |  |
| **True positives** (patients with OSA) | 42 studies 2370 patients | cross-sectional (cohort type accuracy study) | serious^a^ | not serious | not serious | not serious | dose response gradient | 135 (130 to 140) | 271 (261 to 279) | 542 (521 to 559) | ⨁⨁⨁⨁ High^a^ |
| **False negatives** (patients incorrectly classified as not having OSA) |  |  |  |  |  |  |  | 15 (10 to 20) | 29 (21 to 39) | 58 (41 to 79) |  |
| **True negatives** (patients without OSA) | 42 studies 2370 patients | cross-sectional (cohort type accuracy study) | serious^a^ | not serious | not serious | not serious | dose response gradient | 737 (706 to 762) | 607 (582 to 628) | 347 (332 to 359) | ⨁⨁⨁⨁ High^a^ |
| **False positives** (patients incorrectly classified as having OSA) |  |  |  |  |  |  |  | 113 (88 to 144) | 93 (72 to 118) | 53 (41 to 68) |  |

#### Explanations

a. Unclear risk of bias in more than half of the included studies.
